# Erythroderma and extensive poikiloderma – a rare initial presentation of dermatomyositis: a case report

**DOI:** 10.1186/s13256-018-1618-y

**Published:** 2018-03-24

**Authors:** H. M. M. T. B. Herath, B. S. D. P. Keragala, S. P. Pahalagamage, G. H. C. C. Janappriya, Aruna Kulatunga, C. N. Gunasekera

**Affiliations:** 1 National Hospital, Colombo, Sri Lanka; 2 Dermatology, National hospital, Colombo, Sri Lanka

**Keywords:** Dermatomyositis, Poikiloderma, Erythroderma, Poikilodermatomyositis

## Abstract

**Background:**

Dermatomyositis is a humoral-mediated inflammatory myopathy with symmetrical proximal muscle weakness and dermatological manifestations such as Gottron’s papules, heliotrope rash, periungual abnormalities, and flagellate erythema. Erythroderma is a severe and potentially life-threatening dermatological condition with diffuse erythema and scaling involving more than 90% of the skin surface area. Poikiloderma vasculare atrophicans refers to mottled hyperpigmentation and hypopigmentation of the skin with in-between telangiectases and areas of atrophy and is considered a variant of mycosis fungoides. Poikilodermatomyositis is the term given to the condition with poikiloderma and inflammatory myopathy. Only a few cases are reported on erythroderma in dermatomyositis and poikilodermatomyositis. Erythrodermal pattern of dermatomyositis transforming into poikilodermatomyositis is a recognized rare manifestation of dermatomyositis and we could find only one case report in the literature.

**Case presentation:**

A 53-year-old Sri Lankan woman presented with intermittent fever of 5 months’ duration with erythroderma. Later she developed progressive, symmetrical proximal muscle weakness. Following a short course of small dose steroids, erythroderma settled but changed to extensive poikiloderma involving more than 90% of her skin with her face being relatively spared. She had an early heliotrope rash, shawl sign, and Gottron papules. Electromyography and muscle biopsy were supportive of inflammatory myositis and skin biopsy showed evidence of dermatomyositis. Inflammatory markers and muscle enzymes were also elevated. Autoimmune antibodies and myositis-specific autoantibodies were negative. She was started on orally administered prednisolone 1 mg/kg per day with methotrexate 10 mg once a week and had a good response to treatment with resolution of the skin condition and improvement of muscle power. Imaging studies, endoscopies, and tumor markers did not reveal any malignancy.

**Conclusions:**

This case illustrates a rare presentation of dermatomyositis initially presenting as fever, erythroderma, and proximal muscle weakness and later developing poikiloderma involving more than 90% of the skin. It is important to be aware of this rare presentation to avoid misdiagnosis. With the currently available literature it is not possible to conclude that erythroderma is a bad prognostic factor in dermatomyositis or a predictive factor for a malignancy. Patients have a good response to steroids with a combination of immunosuppressants.

## Background

Dermatomyositis is a humoral-mediated inflammatory myopathy with a female to male predominance of approximately 2:1. Typically insidious, gradually worsening, symmetrical proximal muscle weakness with myalgia and mild muscle tenderness is the usual presentation. Gottron’s sign, heliotrope rash, periungual abnormalities, and flagellate erythema are common dermatological features. More uncommon skin manifestations of dermatomyositis described in the literature are ichthyosis, panniculitis, lichen planus-like lesions, porcelain white atrophic scars, vesicle and bullae formation, follicular hyperkeratosis, malakoplakia, and papular mucinosis [1].

Erythroderma or exfoliative dermatitis is a severe and potentially life-threatening dermatological condition with diffuse erythema and scaling involving more than 90% of the skin surface area. It is usually seen in psoriasis, atopic dermatitis, drug hypersensitivity reactions, and more rarely in cutaneous T cell lymphoma. Poikiloderma vasculare atrophicans refers to mottled hyperpigmentation and hypopigmentation of the skin (poikiloderma) with in-between telangiectases (vasculare) and areas of atrophy (atrophicans) [2]. It may be idiopathic or present in connective tissue diseases (like lupus, dermatomyositis, and scleroderma), lymphomas, genodermatoses, physical trauma (radiodermatitis, burns, and freezing), and with certain poisons like arsenic. Poikilodermatomyositis is a rare variant of dermatomyositis. There are only a few cases of erythrodermic dermatomyositis later changing to poikilodermatomyositis reported in the literature.

## Case presentation

A 53-year-old Sri Lankan woman, with diabetes mellitus for 8 years, presented with intermittent fever of 5 months’ duration with a rash. She had lived in Dubai for the last 3 years. Her fever was associated with constitutional symptoms, loss of appetite, and loss of weight. The rash initially started on her trunk and spread to her upper and lower limbs and later to her face within a few days. The rash was erythematous and involved more than 90% of her skin and started to exfoliate after 1 week. She also developed progressive proximal muscle weakness with arthralgia and myalgia and had been bedbound for the last 1 month. There was no history of a photosensitive rash, oral ulcers, Raynaud phenomenon, or arthritis. She had been treated with antibiotics, fluids, and a high protein diet without a response. Following a trial of a small dose of steroids (0.5 mg/kg per day) for 2 weeks, the erythroderma had resolved. She was sent back to her home country, Sri Lanka, afterwards.

**Fig. 1:**
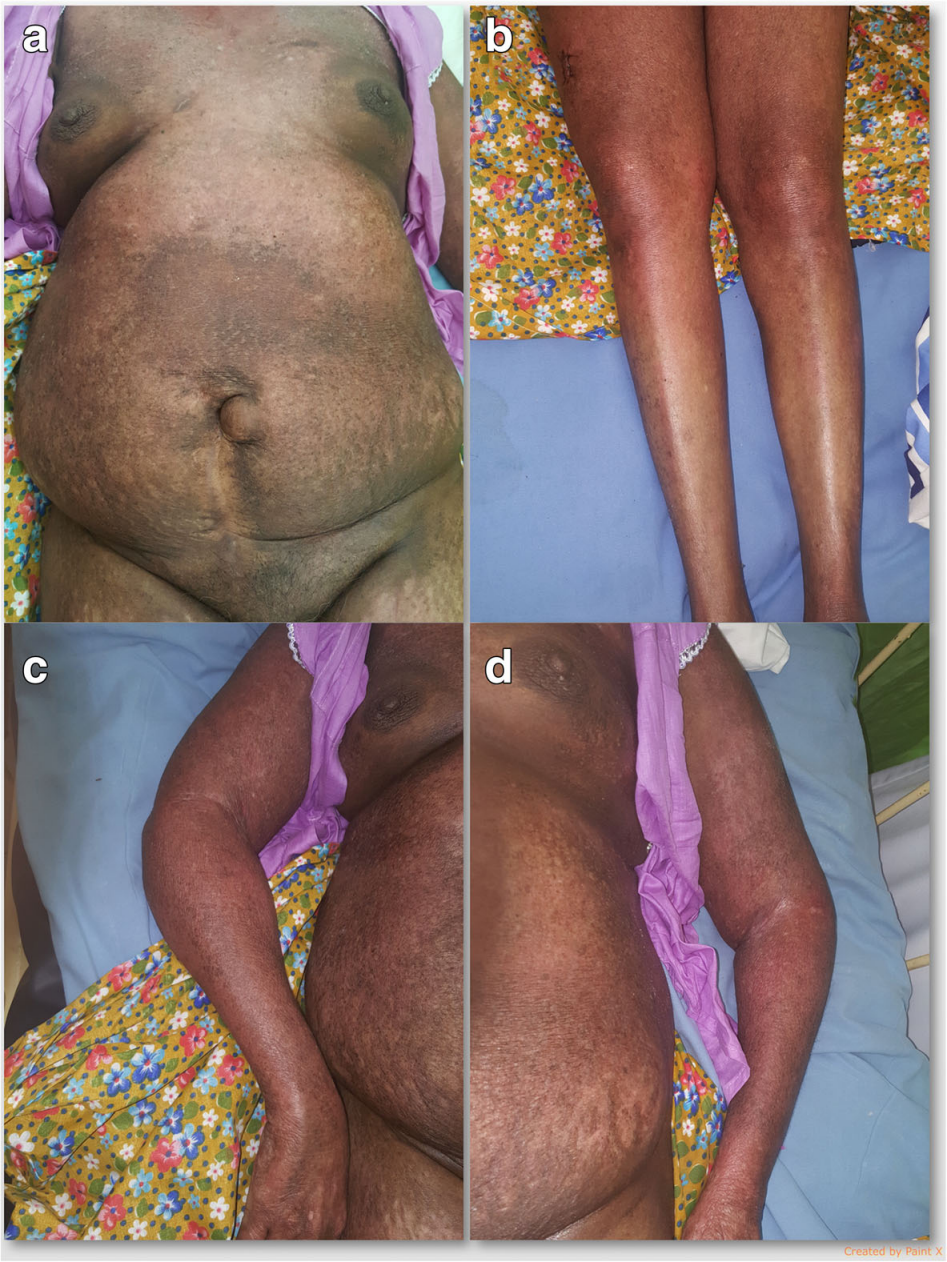
**a** Poikiloderma involving the trunk. **b** Poikiloderma involving the lower limbs. **c** Poikiloderma involving the right upper limb. **d** Poikiloderma involving the left upper limb

**Fig. 2:**
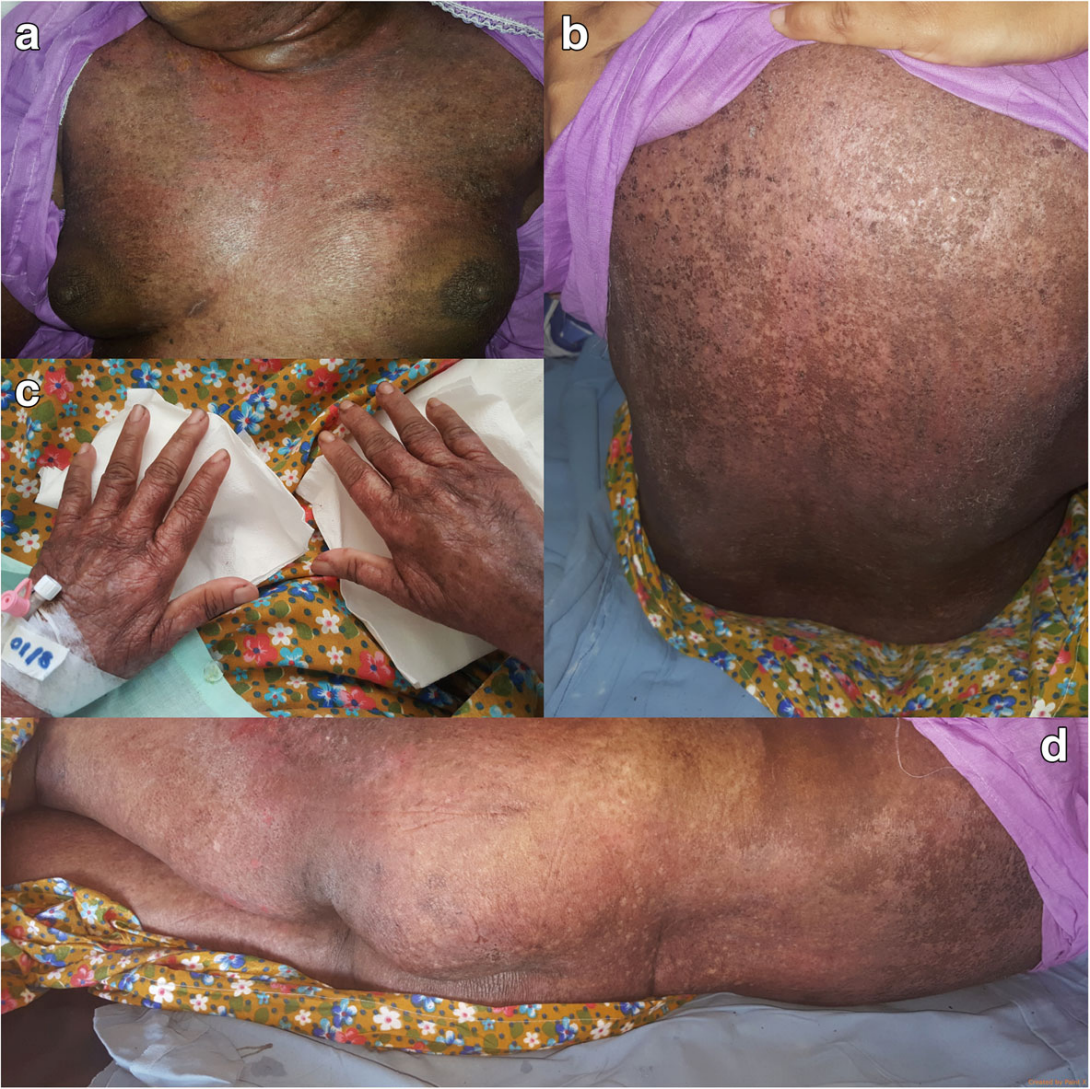
**a** Shawl sign. **b** Poikiloderma involving the back. **c** Dorsum of the hands. **d** Poikiloderma involving the back

**Fig. 3:**
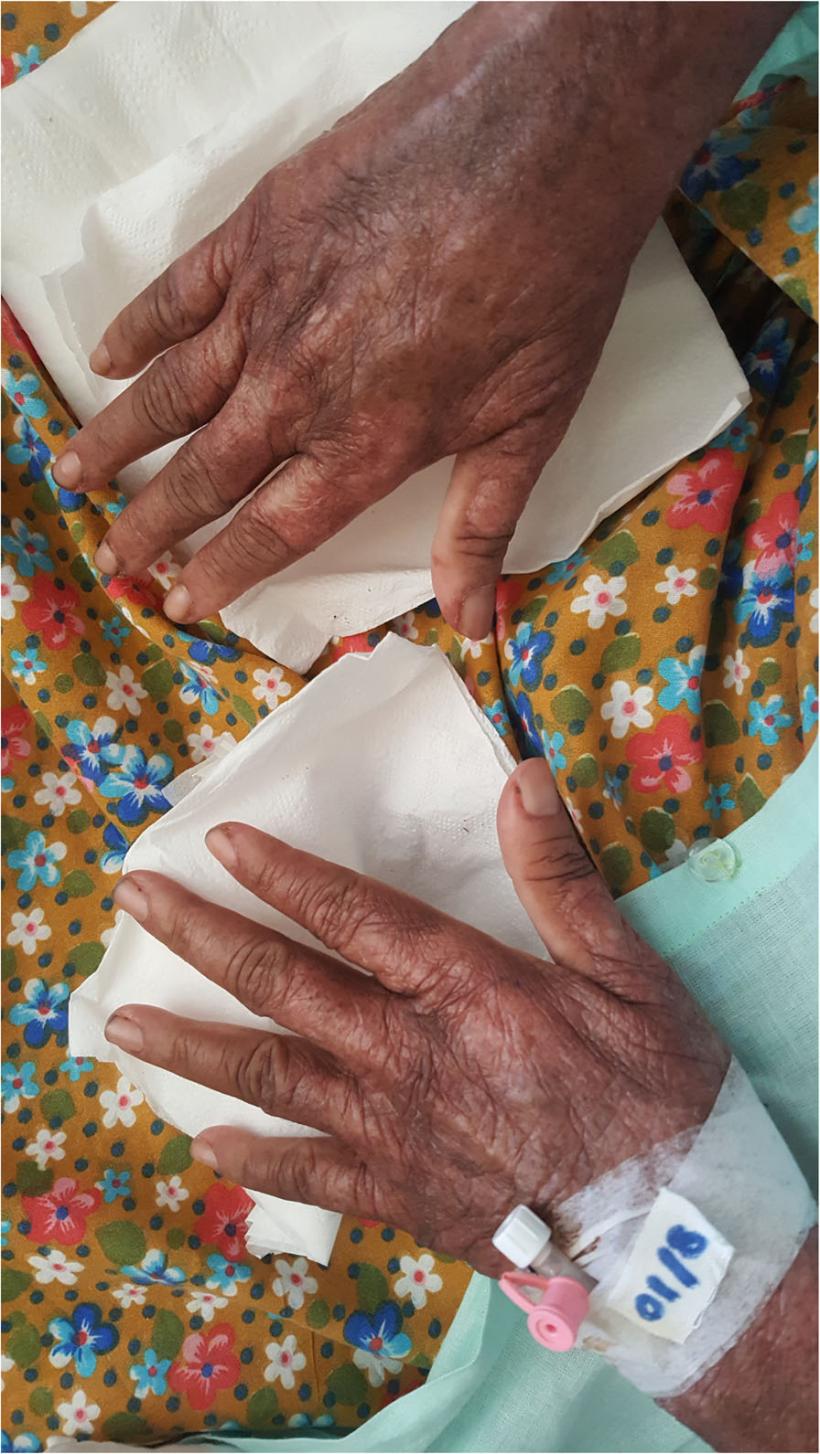
Close look at early heliotrope rash and Gottron papules (difficult to appreciate due to poikiloderma and dark skin)

On admission to our unit she had extensive poikiloderma involving more than 90% of her skin with her face relatively spared. She had an early heliotrope rash, shawl sign and Gottron papules (Figs. 1, 2, and 3). Her proximal muscle power was grade 3 with distal muscle power of 4. Her cranial nerves and eye movements were normal. Respiratory, cardiovascular, and abdominal examinations were normal.

Whole blood count, liver function tests, and renal function tests were normal except for a normochromic normocytic anemia and marginally low albumin (Table 1). Inflammatory markers were elevated with an erythrocyte sedimentation rate (ESR) of 110 in first hour and C-reactive protein (CRP) was 45 mg/L (< 6). Creatine phosphokinase was high at 630 U/L (26–140) and lactate dehydrogenase (LDH) was 712 U/L (225–450). Electromyography showed short, polyphasic, small motor unit potentials with early recruitment. A muscle biopsy from her quadriceps revealed features of inflammatory myopathy including muscle tissue showing focal perivascular atrophy, and some degenerating and necrotic muscle fibers with mild chronic inflammatory cell infiltrates. A skin biopsy from her right upper arm and abdomen revealed a thin epidermis, basal vacuolar degeneration, and mild perivascular lymphatic infiltrates. The papillary dermis showed edema, colloid bodies, pigmentary incontinence, and foci suggestive of mucinous change. Thyroid function tests were normal and she was negative for human immunodeficiency virus (HIV). Autoimmune antibodies were negative including antinuclear antibodies (ANA) and double-stranded deoxyribonucleic acid (DS-DNA). Out of myositis-specific autoantibodies, anti-Jo-1 was negative and antibodies to signal recognition particle and antibodies to Mi-2 were not done. An ultrasound scan of her abdomen and contrast-enhanced computed tomography (CT) of her chest, abdomen, and pelvis did not reveal malignancy. Upper and lower gastrointestinal endoscopy was normal. Cancer antigen (CA) 125, CA 19-9, and carcinoembryonic antibody were within reference ranges. Two-dimensional echocardiography did not show evidence of cardiomyopathy.

**Table 1:** Full blood count, liver function tests, and renal function tests

WBC 5.15 × 103/μL	Neutrophil 86.4%	Lymphocyte 9.7%
Hemoglobin 10.3 g/dL	RBC 3.62 × 10^6^/μL	Platelet 226 × 10^3^/μL
MCV 87.6 fL (80–100)	MCH 28.5 pg (27–34)	MCHC 32.5 g/dL (32–36)
Serum creatinine 1.13 mg/dL	Serum sodium 140 mmol/L	Serum potassium 4.1 mmol
Albumin 34 g/L	Globulin 26.0 g/L	Alkaline phosphatase 66 U/L (30–120)
AST 44 U/L	ALT 19 U/L	INR 1.2
Ionized calcium 1.27 mmol/L (1.0–1.3)	Serum magnesium 1.1 mmol/L (0.8–1.1)	Phosphorous inorganic 4.21 mg/dL (2.5–4.3)

*ALT* alanine aminotransferase, *AST* aspartate aminotransferase, *INR* international normalized ratio, *MCH* mean corpuscular hemoglobin, MCHC mean corpuscular hemoglobin concentration, *MCV* mean corpuscular volume, *RBC* red blood cells, *WBC* white blood cells

**Fig. 4:**
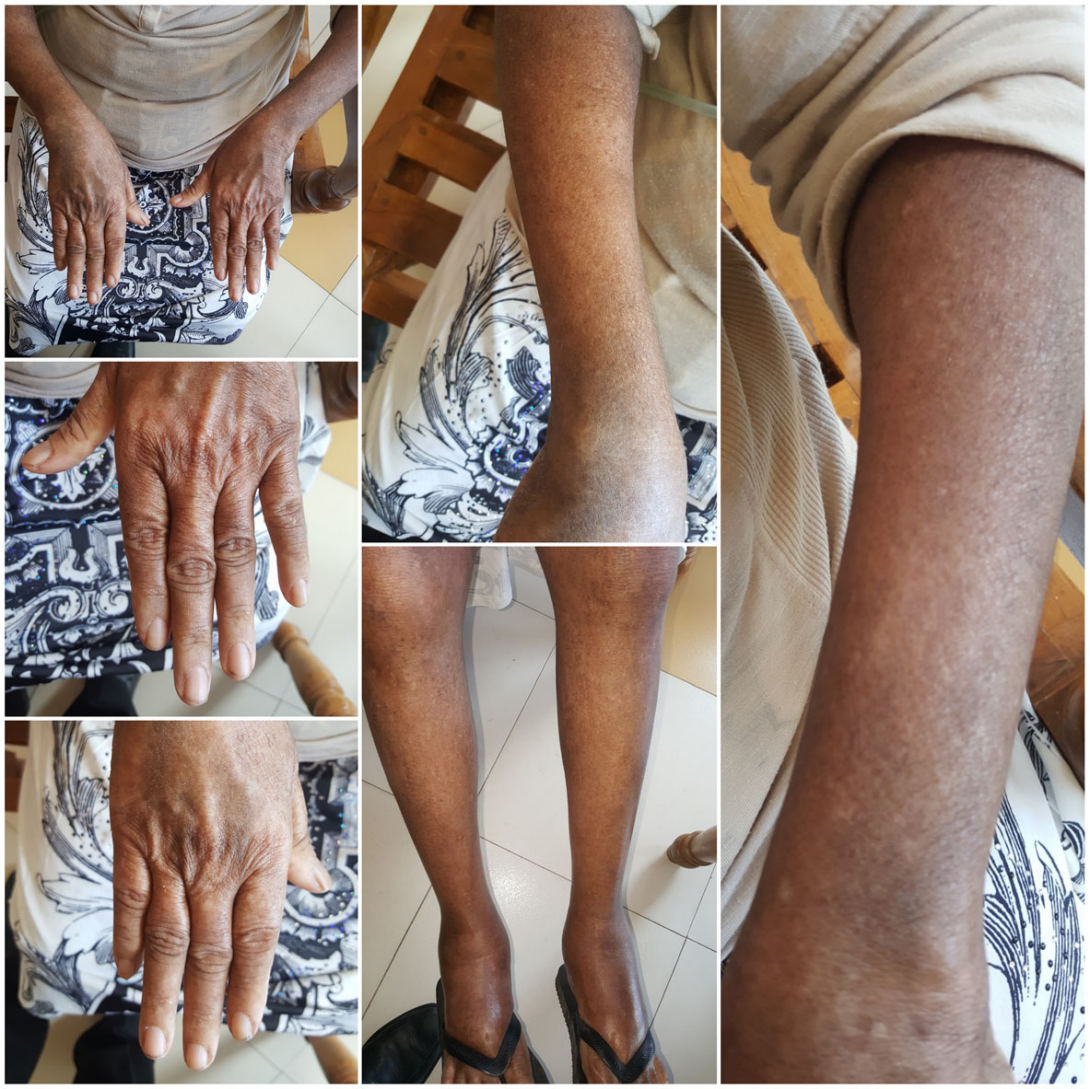
Four weeks following treatment

Due to dysphagia, she was commenced on nasogastric feeds. Physiotherapy was started and early mobilization initiated. Following the diagnosis of dermatomyositis, orally administered prednisolone 1 mg/kg per day was started with methotrexate 10 mg once a week. Calcium supplementations and alendronate were started as bone prophylaxis against osteoporosis. She improved clinically with resolution of skin lesions (Fig. 4) and improving muscle power. She was discharged home from our unit on orally administered prednisolone and methotrexate with a plan to tail off steroids gradually.

## Discussion

In our patient, dermatomyositis was diagnosed based on Bohan and Peter criteria, formulated in 1975 [3]. She had

•Symmetric proximal muscle weakness

•Rash of dermatomyositis (heliotrope rash, Gottron papules, and shawl sign)

•Elevated serum muscle enzymes (creatine phosphokinase)

•Myopathic changes on electromyography [4]

•Characteristic muscle biopsy abnormalities and the absence of histopathologic signs of other myopathies.

It is considered to be a humoral-mediated disorder with the involvement of complements, and cellular infiltrates are seen around blood vessels [5–7]. Erythroderma is a rare condition. There is a complex interaction of cytokines, chemokines, and intercellular adhesion molecules with massive recruitment of inflammatory cells to the skin and increased epidermal turnover resulting in exfoliation. As was seen in our patient, it starts as erythematous patches which increase in size and coalesce into a generalized bright red erythema. Over 90% of the skin is involved and is red, tender, and warm to touch. Scaling is a common feature especially when erythroderma is present for more than 1 week [8, 9]. Unfortunately we do not have photographs of this stage as our patient was in Dubai and the phase of erythroderma had resolved by the time she arrived in Sri Lanka.

Only a few cases have been reported on erythroderma in dermatomyositis [10–17]. Three of them were associated with internal malignancies: gastric cancer [10, 11] and hepatocellular carcinoma [14]. Kim and colleagues described a 90-year-old man with proximal muscle weakness and violaceous to erythematous, confluent, scaly skin lesions involving more than 90% of his total body area [10]. Their electromyography, muscle biopsy, and laboratory investigations were supportive of inflammatory myopathy and gastroendoscopy showed a Borrmann type 1 gastric cancer [10]. Maruani *et al*. reported the case of a 64-year-old patient diagnosed as having dermatomyositis and liver carcinoma with lung metastasis [14]. He presented with erythroderma, epidermal necrosis, and symmetrical proximal muscle weakness [14]. In this article, the authors also mentioned that numerous cases of diffuse erythema were reported in dermatomyositis, which resembled erythroderma. They also stated that it is not possible to set out that erythroderma is a bad prognostic factor in dermatomyositis and in the literature the mortality and frequency of an associated malignancy is the same [11]. Kim *et al*. also supported this in their article and stated that additional evidence is needed to confirm that erythroderma is a predictive factor for a malignancy in patients with dermatomyositis [10]. In our patient too, the screening tests for malignancy were negative.

“Poikiloderma” is a morphologic and descriptive term referring to a combination of cutaneous atrophy, telangiectasia, and varied macular pigmentary changes that lead to mottled skin appearance [18]. In 1906 and 1908 Jacobi described this unusual cutaneous syndrome which he called poikiloderma atrophicans vasculare [19] and much controversy was raised as to whether it is a separate entity or an early or end stage of other skin diseases. Then, in 1906, Petges and Cléjat described a patient with myositis with cutaneous atrophy and poikiloderma and termed the condition “atrophic sclerosis of the skin and generalized myositis” [20]. Petges *et al*. gave the condition the name “poikilodermatomyositis” [21]. Petges and Petges thoroughly reviewed the subject of poikiloderma and distinguished three forms of poikiloderma: (1) a purely cutaneous form; (2) poikilodermatomyositis, a form associated with poikiloderma and generalized myositis; and (3) poikilodermatomyositis accompanied by subcutaneous calcareous concretions [22].

It is a rare condition and only a few case reports are described in literature. Marcus and Wooldridge reported the case of a 23-year-old woman with progressive erythema involving her entire body who later developed proximal muscle weakness, tenderness, and poikiloderma [23]. Bambe described a 10.5-year-old girl with muscle weakness, joint stiffness, Raynaud phenomenon, poikiloderma, and scleroderma-like skin [24]. After that several cases were reported of which the latest was reported by Perales-Martinez *et al*.; Perales-Martinez *et al*. described a female patient who initially had poikiloderma and later developed heliotrope erythema, periorbital edema, Gottron’s papules, progressive proximal muscle weakness, loss of weight, and interstitial lung disease with emphysematous bullae, bronchiectasis, and areas of pulmonary fibrosis [25].

Poikilodermatomyositis was associated with other manifestations in some case reports. Thyresson reported the case of a patient with poikilodermatomyositis with subcutaneous calcification [26]. Marcus and Wooldridge reported the case of a 23-year-old woman with dermatomyositis who later showed features of poikiloderma vasculare atrophicans, scleroderma, panniculitis, periarteritis nodosa, and calcinosis cutis [23]. Other cases had poikilodermatomyositis with subcutaneous calcareous concretions [27, 28], poikilodermatomyositis with multiple calcinosis of the soft tissues [29], poikilodermatomyositis with retinal hemorrhages and secondary glaucoma [30], and severe oligophrenia, amyotrophia, and corneal changes with poikilodermatomyositis [31]. Pedragosa Jove *et al*. reported a case of erythrodermal pattern of dermatomyositis in transit to poikilodermatomyositis [12] similar to our patient.

Our patient made a good recovery with steroid treatment and methotrexate. Up to now screening studies for occult malignancies have been negative.

## Conclusions

This case illustrates a rare presentation of dermatomyositis initially presenting as erythroderma and later developing poikiloderma involving more than 90% of the skin. This case is reported because only a few cases are available in the literature on poikilodermatomyositis with extensive skin involvement and we could find only one case with an erythrodermal pattern of dermatomyositis in transit to poikilodermatomyositis. Our patient was not properly diagnosed for 5 months, which resulted in delayed treatment and severe disability so it is important to be aware of this rare presentation. This case also highlights the importance of a multidisciplinary approach to patients with rare manifestations since a team of physicians, dermatologists, and rheumatologists was involved in the diagnosis. With the currently available literature it is not possible to conclude that erythroderma is a bad prognostic factor in dermatomyositis or a predictive factor for a malignancy. Patients have a good response to steroids with a combination of immunosuppressants with resolution of the skin condition and improvement in muscle power.

## Abbreviations

ANA: Antinuclear antibodies
CA: Cancer antigen
CRP: C-reactive protein
CT: Computed tomography
DS-DNA: Double-stranded deoxyribonucleic acid
ESR: Erythrocyte sedimentation rate
LDH: Lactate dehydrogenase
